# Dose adductor canal block combined with local infiltration analgesia has a synergistic effect than adductor canal block alone in total knee arthroplasty: a meta-analysis and systematic review

**DOI:** 10.1186/s13018-019-1138-5

**Published:** 2019-04-11

**Authors:** Wei Zuo, Wanshou Guo, Jinhui Ma, Wei Cui

**Affiliations:** 1 Peking University China-Japan Friendship School of Clinical Medicine, 2 Yinghuadong Road, Chaoyang District, Beijing, 100029 China; 2 Center for Osteonecrosis and Joint Preserving & Reconstruction, Department of Orthopaedic Surgery, China-Japan Friendship Hospital, 2 Yinghuadong Road, Chaoyang District, Beijing, 100029 China

**Keywords:** Adductor canal block, Local infiltration analgesia, Postoperative analgesia, Total knee arthroplasty

## Abstract

**Background:**

Both adductor canal block (ACB) and local infiltration analgesia (LIA) are effective procedures for postoperative pain control in total knee arthroplasty (TKA) without motor blockade. However, whether ACB combined with LIA has synergistic effect than ACB alone remains unknown. We hypothesized that ACB combined with LIA would have better postoperative pain control, less rescue opioid consumption and faster rehabilitation than ACB alone, without higher adverse event rate.

**Methods:**

We conducted a meta-analysis to identify relevant articles involving ACB + LIA and ACB alone in patients who underwent TKA from online register databases such as PubMed, Medline, Embase, Web of Science, and the Cochrane Library. The primary outcomes were visual analog scale (VAS) score and morphine consumption. Secondary outcomes were postoperative range of motion (ROM) and adverse event rate.

**Results:**

According to the keyword search from online register databases, a total of 879 articles were identified, of which six articles that met the inclusion criteria were determined as eligible. There were three randomized controlled trials (RCTs) and three non-randomized prospective studies. As compared to the ACB alone group, the ACB + LIA group had lower VAS at rest on postoperative day 0 and 1, as well as significantly less morphine consumption on postoperative day 0 and 1 and significantly better postoperative ROM. There were no significant differences in adverse event rate.

**Conclusion:**

As compared to ACB alone, ACB + LIA provides better analgesia and faster functional rehabilitation in patients who underwent TKA.

## Introduction

Total knee arthroplasty (TKA) is a very well-established surgical procedure for patients with end-stage knee osteoarthritis and rheumatoid arthritis. [1] Usually, patients who underwent TKA had intense moderate to severe postoperative pain and difficulty to manage, which seriously affected life quality and postoperative rehabilitation. Effective analgesic regimens have been shown to result in earlier physical therapy and faster recovery leading to better clinical outcomes, shorter hospital stays and less postoperative complications. An effective analgesic regimen for TKA should not only achieve effective analgesia but also preserve muscle strength, which is essential for earlier physical therapy and faster recovery.

Several analgesic regimens including epidural analgesia (EA), femoral nerve block (FNB), patient-controlled analgesia (PCA), adductor canal block (ACB), and local infiltration analgesia (LIA) have been proven to provide effective postoperative pain control for TKA. However, the use of EA and PCA have been associated with various side-effects including urinary retention, pruritus, severe nausea and vomiting. [2, 3] FNB is one of the most commonly used standard postoperative pain relief protocols for TKA, which has proven effective analgesic effect. However, some recent studies reported that it was usually associated with postoperative weakness of the quadriceps, which may increase the risk of falling during postoperative rehabilitation process, thereby hindering early rehabilitation exercises.

Ultrasound-guided ACB, a blockade of the saphenous nerve, medial femoral cutaneous nerve, vastus medialis nerve, medial retinacular nerve and probably articular branches of the obturator nerve, is a relatively new analgesic regimen that was proven to be effective for postoperative pain control without weakening quadriceps muscle strength as compared to FNB. [4–8] However, ACB only provides analgesia to the anterior medial and aspects of the knee capsule, and cannot provide complete pain relief for posterior knee pain. LIA has been introduced as an alternative technique of pain control for TKA with the advantages of no influence on quadriceps strength, ease of performance, effective pain control and low rate of anesthetic systemic complications. [9–12] However, given its disadvantages of the duration and efficacy, LIA alone may be not the best recommendation. [13, 14] Whether LIA combined with peripheral nerve block, especially ACB, has a synergistic effect than ACB alone remains controversial.

## Materials and methods

### Search strategy

This study was conducted in four phases according to the Preferred Reporting Items for Systematic Reviews and Meta-Analyses (PRISMA) Statement reporting guidelines for the meta-analysis of intervention trials. Ethical approval was not necessary since all the data used in this study were extracted from published articles and did not involve any individual personal data. Clinical trials that compared ACB + LIA with LIA alone for postoperative pain control in patients who underwent TKA were identified. Online register databases including PubMed, Medline, Embase, Web of Science, and the Cochrane Library were searched till September 2018. Two authors (ZW and MJH) completed the article search with the help of the librarians. The search terms included “adductor canal block” OR “saphenous nerve block” OR “subsartorial canal block” OR “infrapatellar block” OR “periarticular infiltration” OR “local infiltration analgesia” OR “intraarticular infiltration” AND “total knee arthroplasty” OR “total knee replacement.” Publication language was limited to English. Reference lists of all eligible studies and relevant reviews were manually searched for any additional trials.

### Selection criteria

The selection criteria used for the current meta-analysis are listed below.

The inclusion criteria according to the PICOS criteria were studies including:

1. Population: Patients underwent primary TKA.
2. Intervention: ACB + LIA.
3. Comparator: LIA.
4. Outcomes: The primary outcomes included visual analog scale (VAS) (scale 0–10, where 0 = no pain and 10 = worst imaginable pain) score at rest (8 h, 24 h, 48 h) and rescue opioid consumption (all opioids given were converted to morphine equivalents at 8 h, 24 h, 48 h). The secondary outcomes included postoperative range of motion (ROM) and adverse event rate.
5. Study design: Interventional studies.

The exclusion criteria were studies that were:

1. Case reports.
2. Non-comparative studies or non-human studies.
3. Lacking in scientific design.

### Data extraction

Two authors (ZW and MJH) independently reviewed the full text of the included studies that met the selection criteria. Data including author, publication year, study design, gender, population, age, intervention, type of anesthesia, primary outcomes, and secondary outcomes were extracted. The primary outcomes included visual analog scale (VAS) (scale 0–10, where 0 = no pain and 10 = worst imaginable pain) score at rest (8 h, 24 h, 48 h) and rescue opioid consumption (all opioids given were converted to morphine equivalents at 8 h, 24 h, 48 h). The secondary outcomes included postoperative range of motion (ROM) and adverse event rate. For studies with incomplete or missing data, we contacted the authors to ensure the accuracy of the data.

### Study quality assessment

**Fig. 1:**
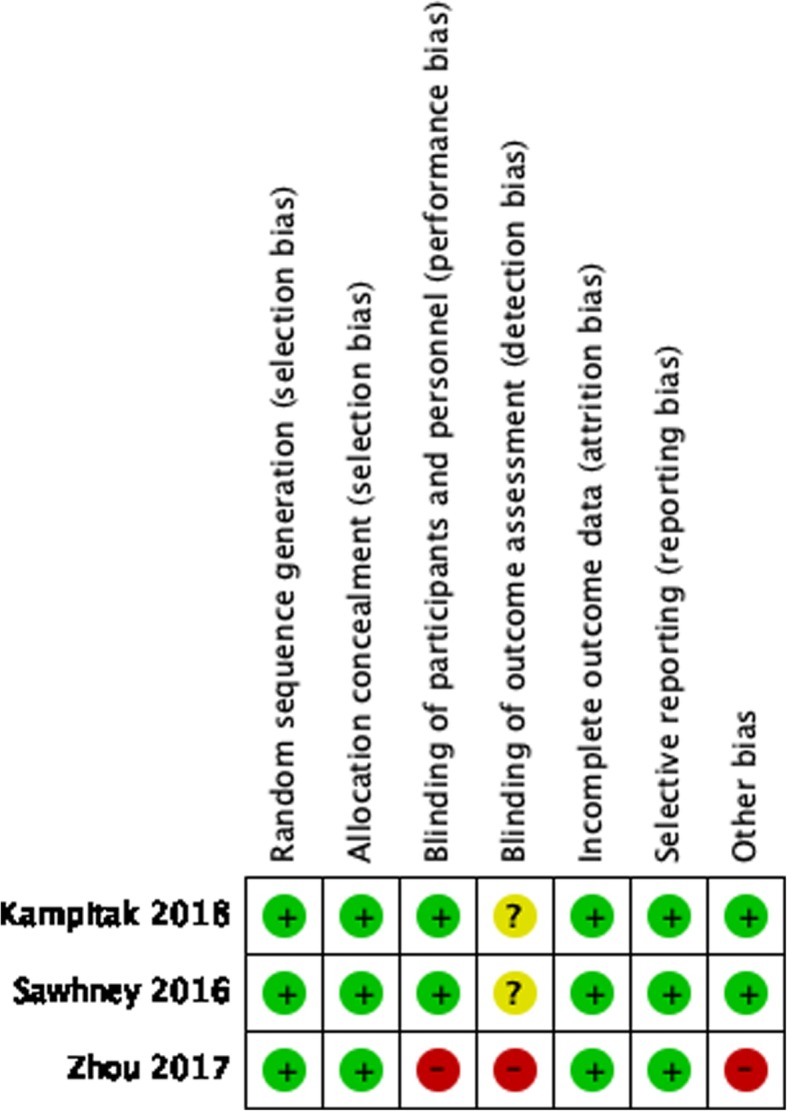
Results of the methodological quality evaluations. Green indicates that the criterion is satisfied. Yellow indicates that it is unclear whether the criterion is satisfied or not. Red indicates that the study did not meet the criterion

The methodological quality of randomized controlled trials (RCTs) were assessed using a modified version of the Jadad Scale (0 [“very poor”] to 7 [“rigorous”]). The Newcastle-Ottawa scale (NOS) (0 [“very poor”] to 9 [“rigorous”]) was used for nonrandomized control trials (nRCTs). The modified version of Jadad Scale includes four domains: randomization, concealment of allocation, double blinding, withdrawals and dropouts (Fig. 1). The NOS-based methodological quality assessment was conducted in three domains: study selection, intergroup comparability and exposure (Table 1). The higher the score, the better was the quality of the article. The assessment was independently performed by two authors, and disagreements were resolved by discussion.

**Table 1:** Newcastle-Ottawa scale

Study	Is the case definition adequate?	Representativeness of the cases	Selection of Controls	Definition of Controls	Comparability of cases and controls on the basis of the design or analysis	Ascertainment of exposure	Same method of ascertainment for cases and controls	Non-Response rate	Scores
Sankineani et al 2018	1	1	1	1	2	1	1	1	9
Sankineani et al 2017	1	1	1	1	1	1	1	1	8
Gwam et al 2016	1	1	1	1	1	1	0	0	7

### Statistical analysis and data synthesis

Calculations of this meta-analysis were performed using Review Manager Software (Revman v5.3, Copenhagen: The Nordic Cochrane Centre, the Cochrane Collaboration). The continuous outcomes including VAS score, rescue opioid consumption and postoperative range of motion were assessed using mean difference (MD) or stand mean difference (SMD) with 95% confidence intervals (CI). The dichotomous outcome (adverse event rate) was assessed using relative risks (RR) with 95% CI. *P*-value < 0.05 was considered to be statistically significant. A funnel plot was used to assess the publication bias of included studies.

### Investigation of heterogeneity

Heterogeneity among the studies was assessed using chi-square test based on the P and I^2^ values. I^2^ > 50% indicated substantial heterogeneity. Therefore, a random effect model was used to assess the outcome. If substantial heterogeneity remained, subgroup analysis was used to interpret the potential source of heterogeneity. Since the importance of inconsistency depends on several factors, interpreting the threshold of I^2^ may be misleading. I^2^ < 50% and *P* > 0.1 indicate that the heterogeneity may not be important, and a fixed effect model was used to evaluate the outcome.

## Results

### Search results

A total of 879 articles were initially identified from online register databases by keyword search, and 823 articles were excluded after primary review of the titles and abstracts. Full text of the remaining 56 articles were evaluated and 50 articles that did not meet the inclusion criteria were excluded. Finally, six articles with a total of 643 patients met the selection criteria and were determined as eligible. There were four randomized controlled trials (RCTs) and two non-randomized pilot studies. All the included articles were in English, and were published between 2016 and 2018. The characteristics of the six included articles are presented in Table 2.

**Table 2:** Characteristics of the included studies

Study, year	No. ACB + LIA/ACB	Male patients (ACB + LIA/ACB)	Age (yr),mean (ACB + LIA/ACB)	Anesthesia	ACB group	ACB + LIA group
Sankineani et al 2018	60/60	38/42	60/61	Spinal	Total of 0.2% ropivacaine 20 ml	Intraoperative LIA with 15 ml of 0.2% ropivacaine in addition to ACB.
Sankineani et al 2017	100/100	80/70	65/67	Spinal	Total of 20 ml of 0.2% ropivacaine	Intraoperative LIA with 60 ml saline solution containing 30 ml of 0.2% ropivacaine, 40 mg ketorolac, 0.5 ml of adrenaline, 4 mg of morphine sulfate in addition to ACB.
Gwam et al 2016	75/52	22/19	63/63	Spinal	Total of 5-10 ml 0.2 to 0.75% ropivacaine	Intraoperative LIA with 50 ml saline solution containing 30 mL of 0.25% bupivacaine, with 1:200,000 parts epinephrine, 8 mg of dexamethasone, 2 mg of morphine, and 30 mg of ketorolac in addition to ACB
Zhou et al 2017	20/20	6/7	66.4 ± 5.8 /67.1 ± 10.2	general anesthesia	Total of 30 ml of 0.375% ropivacaine with 5 μg/ml epinephrine	Intraoperative LIA with 100 ml ropivacaine 2 mg/ml with epinephrine 0.5 ml 1 mg/ml in addition to ACB.
Sawhney et al 2016	50/46	21/20	68.3 ± 9.7/ 66.4 (±9.6)	Spinal	Total of 30 mL of 0.5% ropivacaine	Intraoperative LIA with 110-mL normal saline solution containing 300 mg ropivacaine, 10 mg morphine, and 30 mg ketorolac in addition to ACB.
Kampitak et al 2018	30/30	4/3	69.1 ± 5.36 /72.37 ± 8.02	Spinal	total of 0.5% levobupivacaine 20 mL	Intraoperative LIA with 0.5% levobupivacaine 20 mL, morphine 5 mg, adrenaline 0.3 mg in saline solution in a total volume of 100 mL in addition to ACB.

### Results of meta-analysis

1. VAS score at rest.

**Fig. 2:**
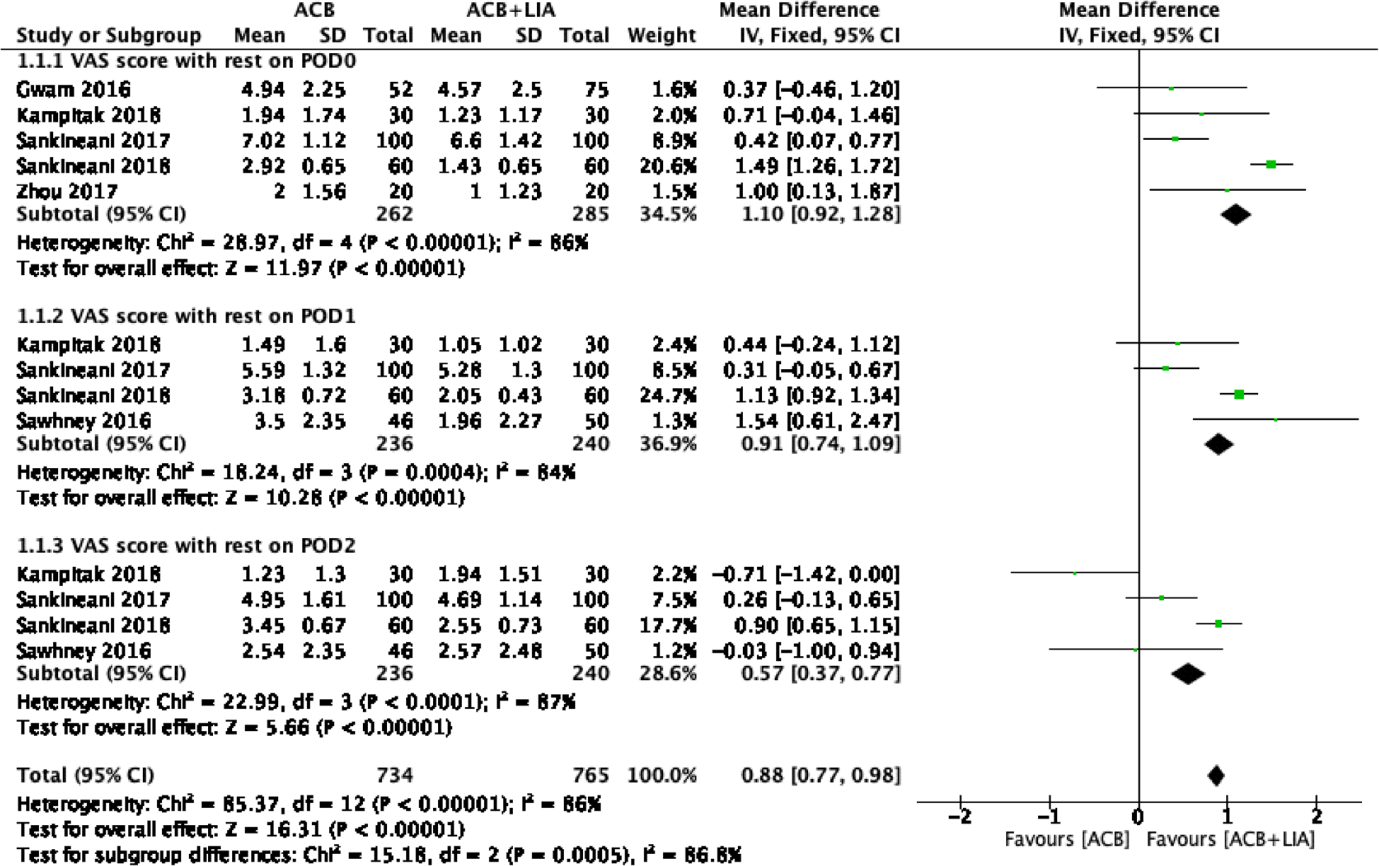
Forest plot analysis of VAS score at rest

Five studies on 547 patients reported the VAS score at rest on postoperative day (POD) 0. The ACB + LIA group was associated with lower VAS score at rest on POD 0 than the ACB alone group (MD = 0.82, 95% CI: 0.22 to 1.42; *P* = 0.007; Fig. 2). Four studies on 476 patients reported the VAS score at rest on POD 1. The ACB + LIA group was associated with lower VAS score at rest on POD 1 than the ACB alone group (MD = 0.81, 95% CI: 0.25 to 1.37; *P* = 0.004; Fig. 2). Four studies on 476 patients reported the VAS score at rest on POD 2. There were no significant differences between the two groups (MD = 0.17, 95% CI: -0.50 to 0.84; *P* = 0.61; Fig. 2).

(2) Morphine consumption.

**Fig. 3:**
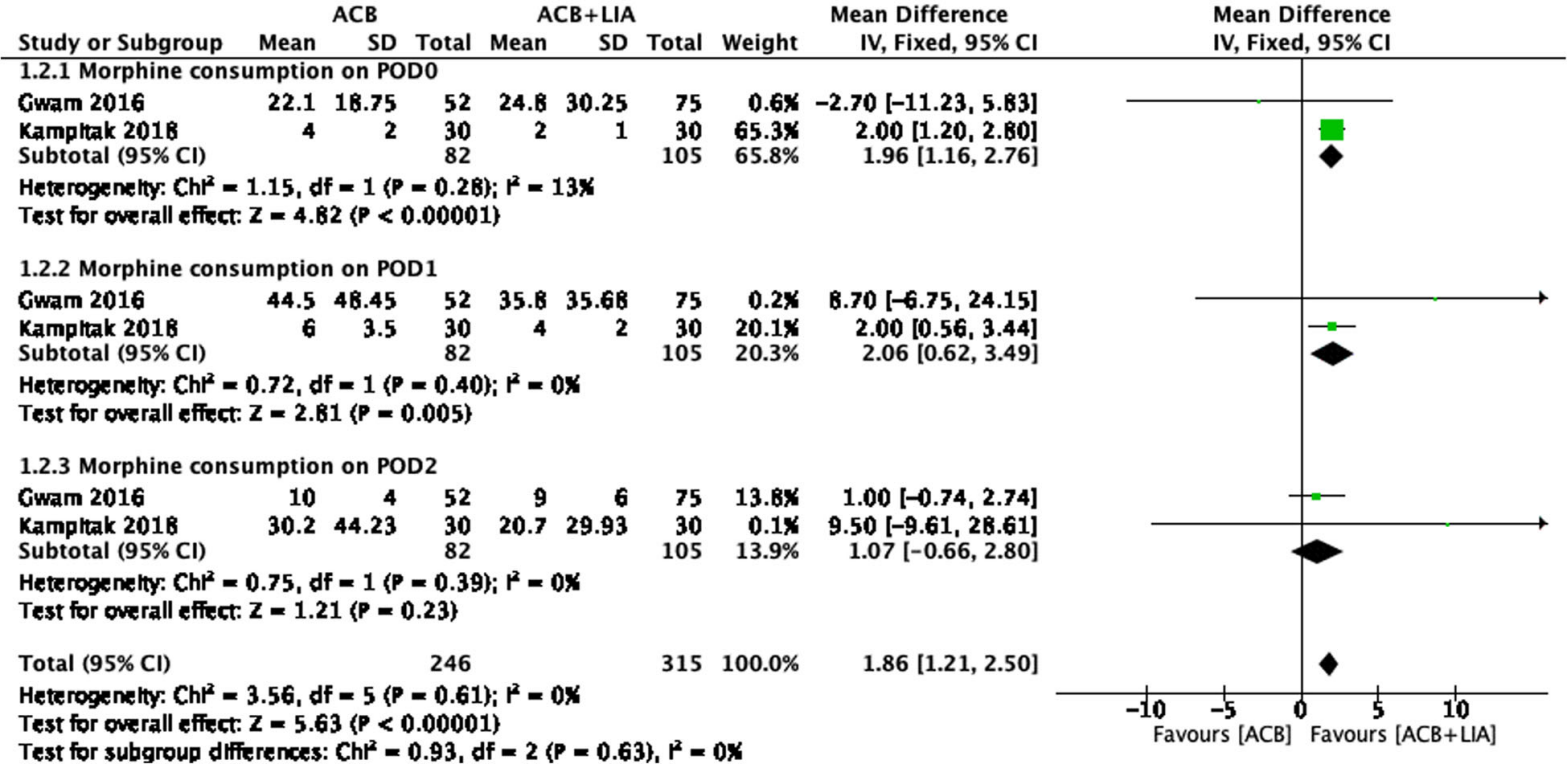
Forest plot analysis of morphine consumption

Two studies on 187 patients reported morphine consumption on POD 0. The ACB + LIA group was associated with less morphine consumption on POD 0 than the ACB alone group (MD = 1.96, 95% CI: 1.16 to 2.76; *P* < 0.05; Fig. 3). Two studies on 187 patients reported morphine consumption on POD 1. The ACB + LIA group was associated with less morphine consumption on POD 1 than the ACB alone group (MD = 2.06, 95% CI: 0.62 to 3.49; *P* = 0.005; Fig. 3). Two studies on 187 patients reported morphine consumption on POD 2. There were no significant differences between the two groups (MD = 1.07, 95% CI: -0.66 to 2.80; *P* = 0.23; Fig. 3).

(3) Postoperative range of motion.

**Fig. 4:**
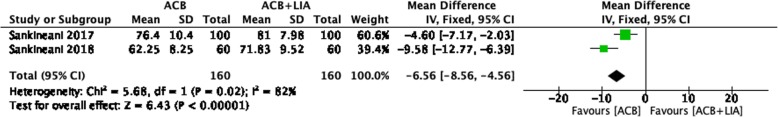
Forest plot analysis of postoperative range of motion

Two studies on 320 patients reported postoperative range of motion. The ACB + LIA group was associated with larger range of motion than the ACB alone group (MD = -6.65, 95% CI: -8.56 to − 4.56; *P* < 0.05; Fig. 4).

(4) Postoperative nausea and vomiting.

**Fig. 5:**
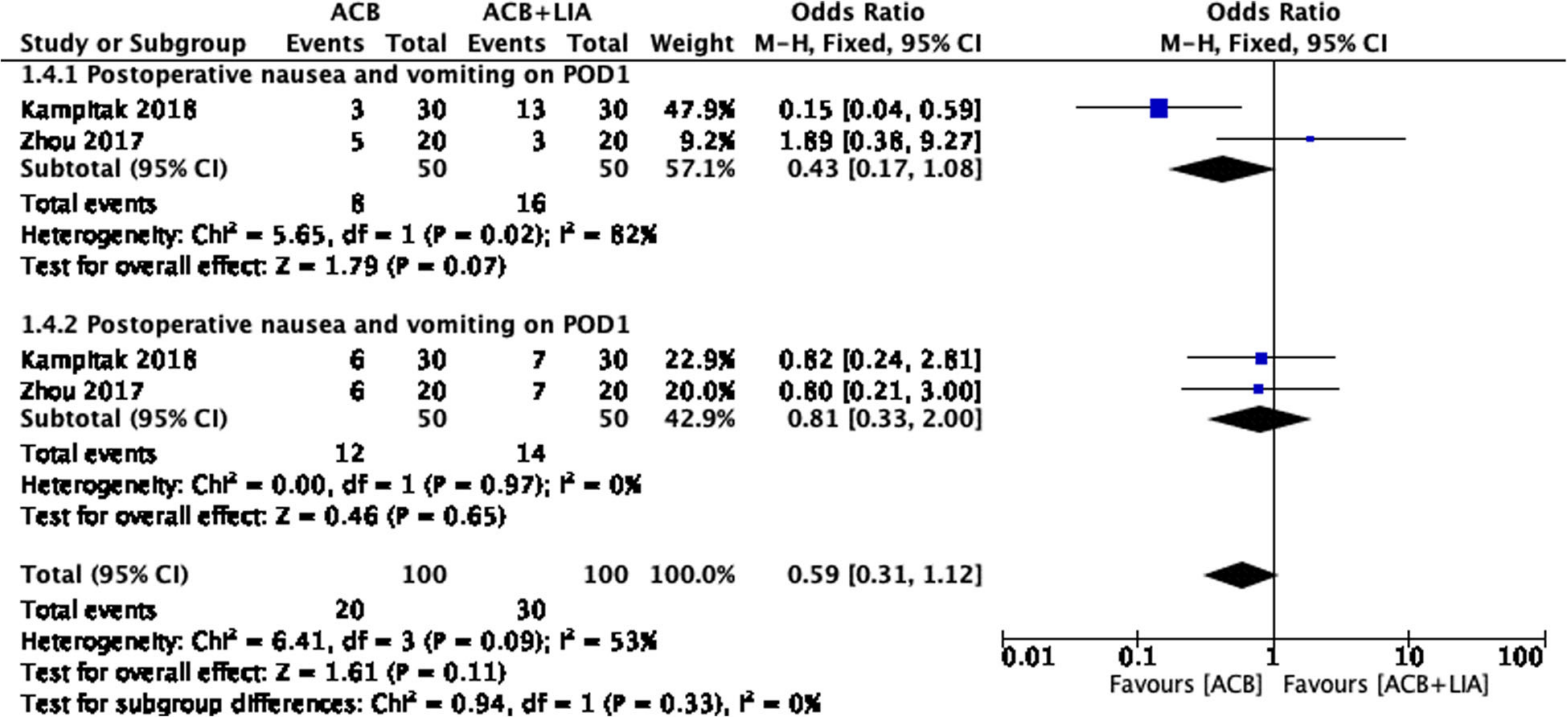
Forest plot analysis of postoperative nausea and vomiting

Two studies on 100 patients reported postoperative nausea and vomiting on POD 0. There were no significant differences between the two groups (MD = 0.43, 95% CI: 0.170 to 1.08; *P* = 0.07; Fig. 5). Two studies on 100 patients reported postoperative nausea and vomiting on POD 1. There were no significant differences between the two groups (MD = 0.81, 95% CI: 0.33 to 2.00; *P* = 0.65; Fig. 5).

## Discussion

Postoperative analgesia is crucial for patients after TKA who usually suffer from early moderate to severe postoperative pain. An ideal postoperative analgesia often contributes to shorter hospital stay, lower complication rates, less opiate consumption and faster recovery [15, 16]. Currently, ACB and LIA are widely used multimodal analgesic regimens with the advantages of preserved quadriceps strength, less complications and adequate pain control, all of which are crucial for safe early ambulation and rehabilitative exercise. A recently published meta-analysis compared ACB and FNB in primary TKA, and found that FNB and ACB had similar pain control after TKA. However, ACB showed better quadriceps muscle strength and mobilization ability, while there were no differences in morphine consumption, patient satisfaction and length of hospital stay [17]. Ma’s meta-analysis found that as compared to LIA alone, ACB + LIA resulted in earlier ambulation, with no significant differences in VAS score, morphine consumption, complication rates and length of hospital stay [14]. Xing’s meta-analysis found that as compared to LIA alone, ACB + LIA had better pain control, less morphine consumption and lower incidence of nausea and vomiting.

The main findings of this meta-analysis were that ACB + LIA had lower VAS score at rest on POD 0 and POD 1, and there were no significant differences on POD 2. Additionally, there was less morphine consumption on POD 0 and POD 1, lower risk of adverse event rates, such as nausea and vomiting, and better postoperative knee range of motion.

With respect to postoperative pain control, this meta-analysis found that the ACB + LIA group had lower VAS score at rest on POD 0 and POD 1. However, this effect of the combination treatment may not have persisted longer than 24 h. These outcomes are consistent with previous studies in which ACB (20–22 h) showed longer duration time than LIA (6–12 h) for postoperative analgesia, and the analgesia effect of LIA decreased with time [6, 18]. Sankineani et al. [19] compared 60 patients each in the ACB + LIA and ACB alone groups, and their results showed that the ACB + LIA group had lower VAS scores on POD 0 and POD 1 with better ROM and ambulatory distance as compared to the ACB alone group. Pain in posterior region of knee was the main complaint in patients of the ACB alone group on POD 0. Sawhney et al. [20] compared ACB + LIA with ACB alone and LIA alone, and their results showed that patients who received ACB + LIA had significantly less pain at rest and walking on POD 1 as compared to patients who received ACB alone. Sankineani et al. [21] compared 100 patients each in the ACB + LIA and ACB alone groups, and the results showed that patients who received ACB + LIA reported significantly lower VAS scores in the immediate postoperative period at 8 h as compared to patients who received ACB alone. However, this effect did not persist longer than 24 h. Zhou et al. [22] compared 20 patients each in the ACB + LIA and ACB alone groups, and their results showed that patients who received ACB + LIA had lower rest and active pain scores 4–8 h post-operation. However, there were no significant differences in the rest and active VAS pain scores between the two groups.

Regarding morphine consumption, this meta-analysis found that the ACB + LIA group was associated with less morphine consumption on POD 0 and POD 1 than the ACB alone group. An RCT by Kampitak et al. [23] also reported that as compared to ACB, ACB + LIA had significant advantages of delaying the time for the first requirement of rescue opioid and less patients requiring rescue opioid during 6 h post-operation. However, these effects might not have persisted beyond 6-8 h since the analgesia effect of LIA decreases with time. Both ACB and LIA had the advantages of not influencing quadriceps strength, which facilitates better ambulation leading to better recovery and rehabilitation of the patient. In the present meta-analysis, we found that the ACB + LIA group was associated with large range of motion than the ACB alone group. However, Kampitak et al. [23] found no difference in Timed Up and Go (TUG) test and quadriceps strength between the ACB + LIA and ACB alone groups at all time points of follow-up.

There were some limitations to this meta-analysis. (1) VAS scores during activity were not analyzed due to inadequate data. (2) Only three of the six included studies were RCTs and others were non-randomized prospective studies, leading to an inherent heterogeneity between the included studies. (3) The small sample size of the included studies resulted in limited statistical power of this meta-analysis. (4) Owing to lack of sufficient extracted data and heterogeneity between the included studies, some of the outcomes could not be analyzed.

## Conclusion

The current meta-analysis found that as compared to the ACB alone group, the ACB + LIA group had lower VAS scores at rest on postoperative day 0 and 1, significantly less morphine consumption on postoperative day 0 and 1 and significantly better postoperative ROM. There were no significant differences in adverse event rates.

## Abbreviations

ACB: Adductor canal block
CI: Confidence intervals
EA: Epidural analgesia
FNB: Femoral nerve block
LIA: Local infiltration analgesia
NOS: Newcastle-Ottawa scale
PCA: Patient-controlled analgesia
POD: Postoperative day
PRISMA: Preferred Reporting Items for Systematic Reviews and Meta-Analyses
RCT: Randomized controlled trial
ROM: Range of motion
RR: Relative risks
SMD: Stand mean difference
TKA: Total knee arthroplasty
VAS: Visual analog scale
